# Universal orthologs infer deep phylogenies and improve genome quality assessments

**DOI:** 10.1186/s12915-025-02328-2

**Published:** 2025-07-28

**Authors:** Md Nafis Ul Alam, Cristian Román-Palacios, Dario Copetti, Rod A. Wing

**Affiliations:** 1 Arizona Genomics Institute, School of Plant Sciences, University of Arizona, Tucson, AZ USA; 2 Plant Biotechnology Laboratory, Department of Biochemistry and Molecular Biology, University of Dhaka, Dhaka, Bangladesh; 3 College of Information Science, University of Arizona, Tucson, AZ USA; 4 Center for Desert Agriculture, Biological and Environmental Sciences and Engineering Division (BESE), King Abdullah University of Science and Technology (KAUST), Thuwal, 23955-6900 Saudi Arabia

**Keywords:** Phylogenomics, Genomics, Gene annotation, Monophyly, Assembly completeness, Assembly quality, Assembly evaluation, Syntenic distance, Collinearity, Broad phylogeny

## Abstract

**Background:**

Universal single-copy orthologs are the most conserved components of genomes. Although they are routinely used for studying evolutionary histories and assessing new assemblies, current methods do not incorporate information from available genomic data.

**Results:**

Here, we first determine the influence of evolutionary history on universal gene content and find that across 11,098 genomes of plants, fungi, and animals comprising 2606 taxonomic groups, 215 groups significantly vary from their respective lineages in terms of BUSCO (Benchmarking Universal Single Copy Orthologs) completeness. Additionally, 169 groups display an elevated complement of duplicated orthologs, likely from ancestral whole genome duplication events. Secondly, we investigate the extent of taxonomic congruence in broad BUSCO-derived phylogenies. For 275 suitable families out of 543 tested, sites evolving at higher rates produce at most 23.84% more taxonomically concordant, and at least 46.15% less terminally variable phylogenies compared to lower-rate sites. We find that BUSCO concatenated and coalescent trees have comparable accuracy and conclude that higher rate sites from concatenated alignments produce the most congruent and least variable phylogenies. Finally, we show that undetected, yet pervasive BUSCO gene loss events lead to misrepresentations of assembly quality. To overcome this, we filter a Curated set of BUSCOs (CUSCOs) that provide up to 6.99% fewer false positives compared to the standard search and introduce novel methods for comparing assemblies using gene synteny.

**Conclusions:**

Overall, we highlight the importance of considering evolutionary histories during assembly evaluations and release the phyca software toolkit that reconstructs consistent phylogenies and offers more precise assembly assessments.

**Supplementary Information:**

The online version contains supplementary material available at 10.1186/s12915-025-02328-2.

## Background

High-quality reference genomes are becoming available for earth’s flora and fauna at an accelerating rate. According to successive annual updates, 7845 new organism genomes were released over the most recent annum by NCBI (National Center for Biotechnology Information) alone [1, 2]. With advancements in long-read sequencing, nuclear conformation capture and optical mapping, the reconstruction of high-quality telomere-to-telomere assemblies [3, 4] is now becoming routine across all extant clades in the tree of life [5].

Conserved single-copy orthologs are used to create phylogenies [6] and evaluate the completeness of new assemblies [7], yet current tools and databases remain mostly oblivious to their varying evolutionary histories and taxonomic biases. For instance, OrthoDB [8] is an established database of universal orthologs, but does not specifically explore the genome-wide variations in gene presence within major taxonomic groups. Similarly, OrthoFinder [9] is used to reconstruct gene trees and species phylogenies, but does not analyze phylogenetic conflicts within and between gene features in alignment sites. Moreover, the detrimental effects of disregarding information about evolutionary history when using universal orthologs for assembly completeness tests [10] has been overlooked in available methods [7]. Hence, a systematic exploration of public genomic data has the potential to improve existing methods for the utilization of universal orthologs in phylogenomics and assembly quality assessments.

Universal single-copy orthologs are the most stable components of genomes as they remain identifiably conserved in higher eukaryotes that diverged over millions of years ago [11]. A query set of universal single-copy orthologs or BUSCOs (Benchmarking Universal Single-Copy Orthologs) [7] serves as a standard method for benchmarking gene content in newly assembled genomes. Fluctuations in BUSCO gene incidence is seen in some taxonomic groups [10] but the full extent of BUSCO gene absence across genomically well-represented lineages has not been the subject of a focused or recent study. Although these genes remain under an evolutionary constraint of being maintained as single copies to balance dosage, polyploids [12] and descendants of recently genome duplicated ancestors [13–15] carry fractionally elevated copy numbers. As such, copy number variations of BUSCO genes have not been cataloged in detail across taxonomic groups or by gene identity.

BUSCO gene sets have been the basis for some deep molecular phylogenies [6, 16]. BUSCOphylo [17] allows users to create BUSCO phylogenies, but it is not computationally feasible for gigabase-scale genomes or a large number of taxa. It also does not explore the accuracies or inconsistencies of BUSCO-derived phylogenies. Moreover, from the perspective of molecular phylogenetics, while substitution models have been trained on empirical sequences [18–20] to improve likelihood estimates, there have been limited efforts in incorporating divergent reference genome data [21] to derive improved inferences. Among many unknowns, there are known sources of model inadequacies that violate basic phylogenetic assumptions. For instance, gene histories are often obscured by incomplete lineage sorting [22], horizontal gene transfer [23] or hybridization [24] and sites in gene alignments may support conflicting histories due to alignment errors [25], recombination, long-branch attractions [26] or node-density artifacts [27]. Furthermore, alignment concatenation has been shown to be statistically inconsistent for tree reconstructions [28]. This has led to many researchers assaying both concatenated and coalescent trees [18, 29]. Therefore, further research on empirical phylogenomic methods is required to evaluate and improve existing protocols and to promote consistency.

In this study, we compiled BUSCO statistics for all plant, fungal, and animal genomes cataloged in NCBI Genome [30] up to January of 2024. Our objective was to improve methods for the utilization of BUSCO genes in phylogenomics and genome completeness evaluations. Under a wide range of rate and site configurations, we assessed the capacity of BUSCO genes in reconstructing taxonomically congruent phylogenies. We tested individual trees for taxonomic concordance, and tree distributions under the same conditions for variations in terminal leaf bifurcations. Through the constructed BUSCO database, we identified pervasive ancestral gene loss events and provided evidence for 2.25 to 13.33% mean lineage-wise gene misidentifications using the most widely used default BUSCO search parameters. Categorically, we procured a Curated set of BUSCO orthologs (CUSCOs) that attains a higher specificity for 10 major BUSCO eukaryotic lineages, namely Viridiplantae, Liliopsida, Eudicots, Chlorophyta, Fungi, Ascomycota, Basidiomycota, Metazoa, Arthropoda, and Vertebrata. For robust comparisons and evaluations of closely related assemblies, a syntenic BUSCO metric was derived that offers higher contrast and better resolution than standard BUSCO gene searches. Our results and data have been made available through a public database [31] and source code for the phyca software is available through GitHub [32].

## Results

### BUSCO gene content is influenced by evolutionary history

**Fig. 1:**
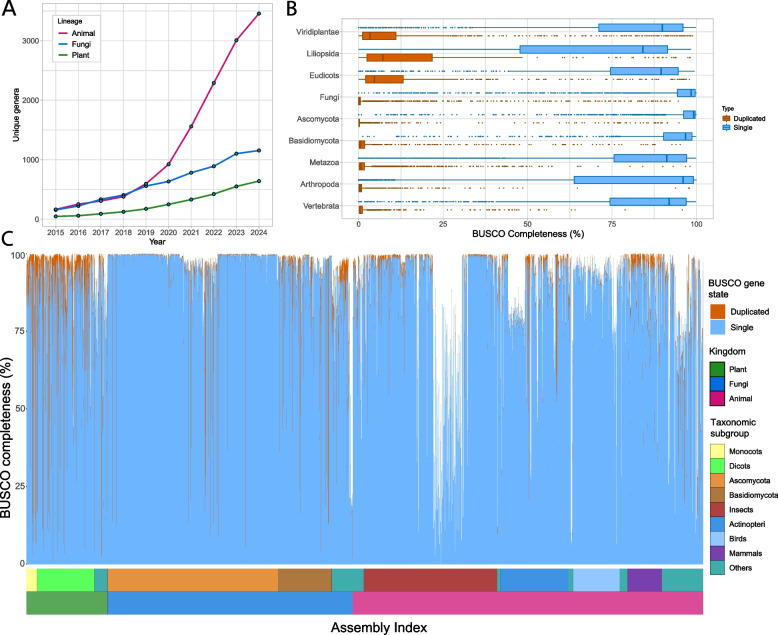
BUSCO database statistics.** A** Genome assemblies for new genera and species are growing linearly for plants and fungi and rapidly for animals, especially in recent years. **B** BUSCO statistics vary for plants, fungi, and animals. The fraction of single-copy and duplicated genes are complementary. More duplications are observed in plants and less variation is notable for the fungi. **C** Some taxonomic groups, such as ascomycetes and insects, are better represented in NCBI genome. Assemblies from bulk genome sequencing projects with relatively low cost per genome appear as a stretch with lower BUSCO completeness. Duplicated fractions are more prominent in plants owing primarily to higher duplication rates and greater incidence of polyploidy

We compiled 11,098 eukaryotic genome assemblies from NCBI and observed that genomes for new animal genera were being released at a greater rate than plants and fungi (Fig. 1A). The majority of NCBI genome assemblies contained a complete or near-complete complement of single and duplicated BUSCO genes (Fig. 1B). Plant lineages had a much higher mean BUSCO duplication rate at 16.57% compared to fungi and animals at 2.79 and 2.21% respectively (Fig. 1B and C). It is known that genomes of higher ploidy are often assembled into variable sets of pseudomolecules [12, 33] and this is reflected in our database (Additional file 1: Figure S1). The mean number of observed copies for the complete BUSCO gene set had 99.05% linear correlation with the number of copies of pseudomolecules in phased and partly phased assemblies (Additional file 1: Figure S1). There were 169, 165, and 258 taxonomic groups out of 2606 total that had significantly elevated means for duplicated BUSCO genes, mean BUSCO copy numbers, and log assembly size respectively (Additional file 2: Table S1). For example, among the well-represented fungal classes, all 13 assemblies of the family Backusellaceae had duplicated BUSCOs significantly greater than other fungal groups with a minimum of 11.42% and mean of 12.18%. For the 25 assemblies in the Mucoraceae family, the minimum and mean for duplicated BUSCOs were 5.1 and 6.54% respectively. The assembly counts, mean, minimum, and maximum number of BUSCO metrics for every taxonomic group including Mann–Whitney *U* test *p*-values for deviation from group means are provided in Additional file 2: Table S1.

Extended drops in BUSCO completeness in Fig. 1C are a result of bulk genome sequencing projects that resulted in large numbers of draft genome assemblies, e.g., Ellis et al. [34] who submitted 822 de novo butterfly genomes, Ronco et al. [35] who submitted 539 cichlid fish genomes. Some taxonomic groups do show a predisposition to comparatively lower BUSCO completeness, as outlined in Additional file 2: Table S1. For instance, a number of *Incertae sedis* fungi-like organisms (mostly microsporidia) were found to contain < 25% BUSCO genes and are seen as a dip at the trail of the fungal bars in Fig. 1C (Additional file 2: Table S1). In terms of taxonomy, it was found that across all BUSCO lineages and taxonomic levels, 215 groups had significantly different mean BUSCO completeness. The complete database, along with taxonomic classifications, assembly and BUSCO statistics are available to download and view at the database website [31].

### Sites evolving at higher rates produce more taxonomically congruent phylogenies

From our compiled data, we sought to determine the best way to utilize BUSCO genes to create broad genome-scale phylogenies spanning large evolutionary distances. Individual phylogenies were tested for agreement with NCBI taxonomic classifications. To assess taxonomic congruence, we created 3566 phylogenetic trees for the 5 largest BUSCO lineages in terms of assembly and gene count. Our tests were focused on the Eudicots, Ascomycota, Basidiomycota, Arthropoda, and Vertebrata lineages. Gene alignments for divergent taxa varied significantly based on parameters passed to the alignment algorithm (Additional file 1: Figure S2). Different lineages had different rate profiles for aligned sites (Additional file 1: Figure S3). Algae, fungi, and early diverging metazoans displayed greater site heterogeneity in their alignments (Additional file 1: Figures S3).

**Fig. 2:**
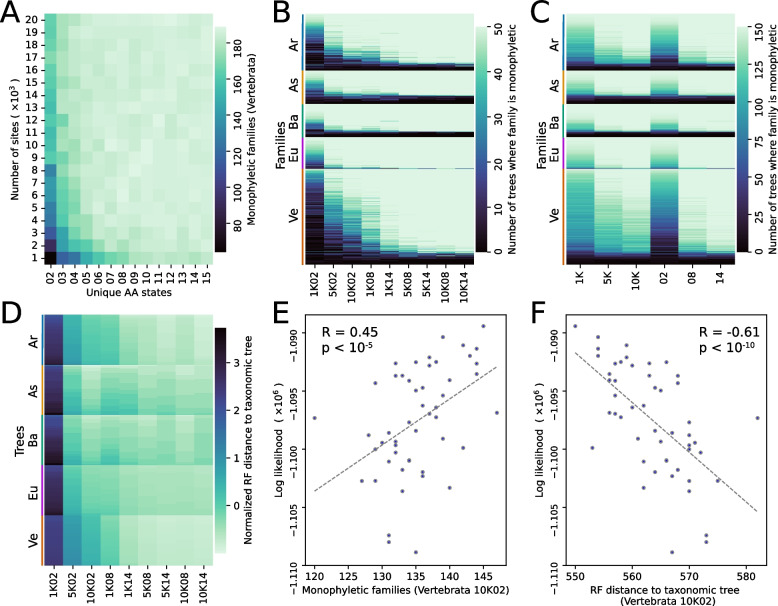
Higher rates are more informative and produce better phylogenies overall.** A** Taxonomic concordance across 13 rate profiles and 20 alignment lengths for the vertebrata lineage. Sites evolving at higher rates and longer alignments share more agreement with taxonomic groupings. **B** Ve, Eu, Ba, As, and Ar represent Vertebrata, Eudicots, Basidiomycota, Ascomycota, and Arthropoda lineages respectively. Along the horizontal axis, 1K, 5K, and 10K represent alignment lengths in kilobases and 02, 08, and 14 represent the number of unique amino acid characters or rate categories for alignment columns. Each row in the heatmap is a unique family. The depth of color is the number of times a family appeared monophyletic out of 50 total trees in each cell. With few exceptions, families are more likely to be found monophyletic at greater rates and sites. **C** Increasing rates have a greater effect on tree concordance relative to increasing sites. **D** Sets of 50 trees are shown on the vertical axis for each lineage and 9 tested conditions are shown on the horizontal axis. The depth of color represents normalized Robinson-Foulds (RF) distance to taxonomic trees. RF distances were normalized by lineage. Across 9 highlighted conditions, a pattern similar to family monophyly counts is generated by RF distances. **E**, **F** Under optimum tree search conditions, tree likelihoods correlate with taxonomic agreement measured by both monophyly counts and RF distances

Phylogenetic trees under different evolutionary rates and alignment lengths were compared for taxonomic congruity. Variations of the LG (Le-Gascuel) [36] and JTT (Jones-Taylor-Thornton) [37] substitution models [36] with different rate categories were consistently found to have the highest likelihood under all conditions (Additional file 3: Table S2). The top 5 best substitution models based on Bayesian Information Criterion (BIC) for each condition with model comparison metrics are included in Additional file 4: Table S3. When the number of unique amino acid residues in an alignment column was used as a proxy for site evolutionary rate, sites evolving at higher rates together with longer alignments generally produced more taxonomically concordant trees (Fig. 2A and Additional file 1: Figure S4). Taxonomic concordance was predominant in eudicots with either 68 or 69 out of 69 total families (98.55–100%) being reconstructed as monophyletic above 4000 alignment length and 5 or more unique amino acids. In arthropods and vertebrates, up to 113 out of 125 (90.40%) and 187 out of 225 (83.11%) respectively were reconstructed as monophyletic. In ascomycetes and basidiomycetes, only up to 60 out of 97 (61.86%) and 63 out of 88 (71.59%) respectively were found monophyletic in any single condition. The lineage and condition-wise monophyly counts are presented in Additional file 1: Figure S4. For each lineage, a consistent number of families were resolved as monophyletic in most of the trees, while some families precariously only appeared monophyletic at certain conditions (Additional file 5: Table S4). Alignments with greater numbers of sites and unique residues almost always resolved greater numbers of families (Fig. 2B). Rate effects were more potent than alignment length (Fig. 2C). Higher taxonomic congruence suggested by family monophyly counts was supported by Robinson-Foulds distances (RF distance) to respective taxonomic trees (Fig. 2D, Additional file 1: Figure S5). It was found that 32 families out of 543 total were monophyletic under all tested conditions. Of the remaining 511, 59.47%, 84.61%, and 86.53% were monophyletic when reconstructed with 2, 8, and 14 (low, moderate, and high) unique amino acids per column respectively and 67.18%, 80.12%, and 83.32% were reconstructed as monophyletic with 1000, 5000, and 10,000 alignment lengths respectively. Under conditions where the alignments did not provide sufficient information to accurately resolve tree topology (Additional file 1: Figure S6), likelihoods correlated positively with family monophyly counts and negatively with RF distances to taxonomic trees (Fig. 2E and F). Variations in taxonomic concordance receded with increasing site counts and evolutionary rates in eudicots, arthropods, and vertebrates, but the pattern was less prominent in ascomycetes and basidiomycetes (Additional file 1: Figure S7). To interpret the relationship between tree likelihoods and taxonomic concordance, we recomputed likelihoods for all trees under a fixed set of alignments. Correlations between mean tree likelihood and taxonomic concordance diminished with longer alignments and faster evolving sites (Additional file 1: Figure S6). At the same time, tree topologies were more stable at the terminal taxa for all lineages at higher evolutionary rates and greater site counts (Additional file 1: Figure S8).

We observed that all five tested lineages showed a similar trend where 462 out of 543 families were found monophyletic at the most informative condition with 14-character columns and an alignment length of 10,000 (Fig. 2B and Additional file 5: Table S4). Of the remaining 81, 42 families could not be resolved as monophyletic (0 out of 50 trees) and the monophyly status of the remaining 39 families remained inconsistent. Rate preferences for monophyly in the queried families were not observed. The Petroicidae family of birds was the only family that yielded monophyletic trees across all 50 trees at rate condition 8, but was not consistently monophyletic in the higher rate condition of 14 with monophyly in 49 out of 50 trees in alignments of length 10,000 (Additional file 5: Table S4).

### Site-filtered concatenation trees have comparable accuracy to coalescent trees

**Fig. 3:**
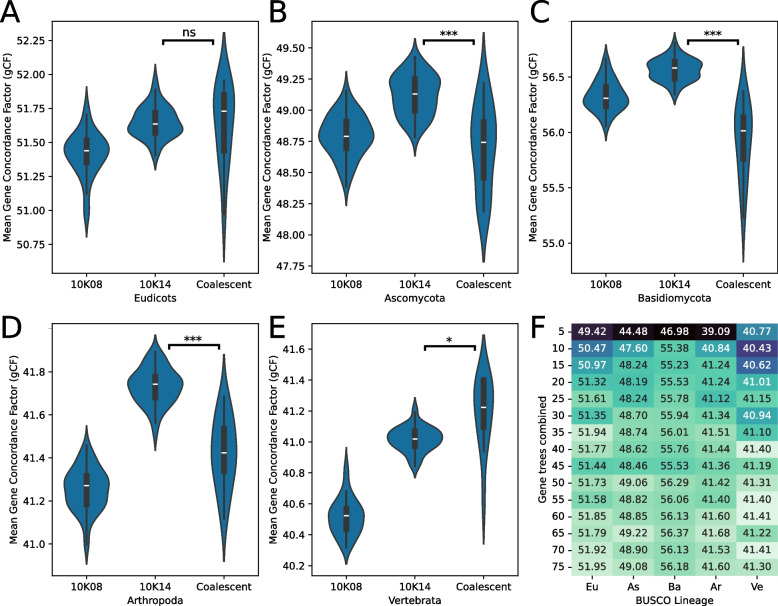
Site-filtered concatenation trees have comparable accuracy to coalescent trees.** A–E** Comparisons of tree sets produced using 10,000 sites of rates 08 and 14 (10K08 and 10K14) to a set of 13 coalescent trees produced from 20 to 75 BUSCO gene trees using mean gene concordance factors (gCF). Welch *t*-test reveals no significant difference in means between 10K14 and coalescent trees for eudicots, higher means in 10K14 for ascomycetes, basidiomycetes and arthropods and a marginally lower mean in 10K14 for vertebrates. ns: not significant; *: 0.01 < *p*-value < 0.05; ***: *p*-value < 0.001. **F** Coalescent trees produced with 5, 10 and 15 gene trees have relatively lower gene concordance than those produced from 20 or more gene trees

We compared sets of trees created from 10,000 sites of 14 unique characters to BUSCO coalescent trees inferred from 20 or more gene trees to compare the two methods. A set of 100 BUSCO gene trees were used to calculate gene Concordance Factors (gCF) along the branches of each individual tree within the two sets of trees from each method. Estimated gCF values for sets of trees with 10,000 sites of 8 unique characters were also illustrated in Fig. 3A–E. In terms of gCF along branches, there were no significant differences between the means of the eudicot trees (Welch’s *t*-test for unequal variances *p*-value 0.85; Fig. 3A). For ascomycetes, basidiomycetes, and arthropods, the site-filtered concatenation trees had significantly higher means than the coalescent trees with Welch *t*-test *p*-values 0.7 × 10^−3^, 1.38 × 10^−5^, and 1.29 × 10^−5^ respectively (Fig. 3B–D). The coalescent vertebrate tree set had a marginally higher gCF mean than the concatenated trees with a Welch *t*-test *p*-value of 0.02 (Fig. 3E). In terms of family monophyly, no variations in taxonomic agreement between concatenated trees and trees created under the multispecies coalescent model were observed (Additional file 1: Figure S9A-E). Coalescent trees based on 20 or fewer gene trees had relatively lower gene concordance and taxonomic agreement compared to coalescent trees created from greater numbers of gene trees (Fig. 3F and Additional file 1: Figure S9F).

### Clade-specific BUSCO gene loss events are pervasive in tested lineages

**Fig. 4:**
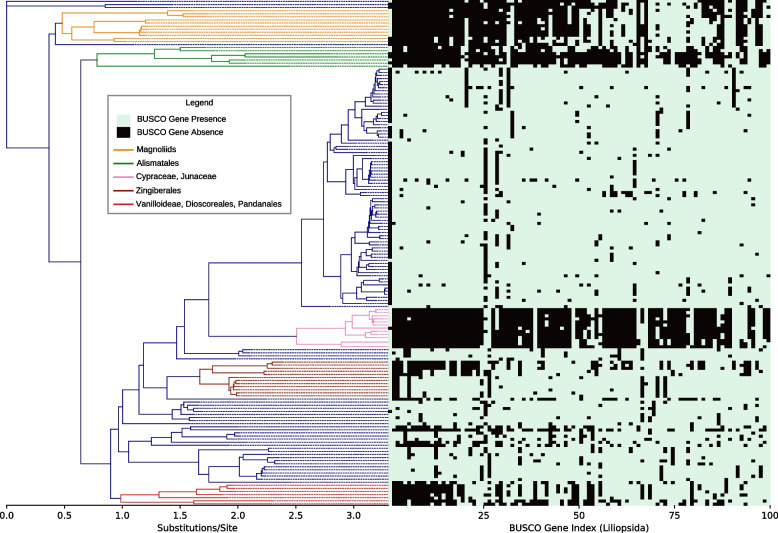
Ancestral BUSCO gene loss events in the Liliopsida clade. On the phylogeny, 164 monocot species are shown. Each column on the grid represents a BUSCO gene within the lineage Liliopsida. Genes are ordered along the horizontal axis based on the frequency at which they were absent in clades containing 3 or more species. Specific gene absence is frequently observed in all or most descendants of some lineages, suggesting an ancestral gene loss event

Absence of a gene in a taxon or clade prevents its inclusion on a gene tree. To assess the relationship between evolutionary history and the absence of BUSCO orthologs, we analyzed BUSCO gene loss events along our phylogenies. Phylogenies for each lineage were created at either specific or generic levels depending on the number of genomes in each lineage (Table 1). Across the 10 lineages, 13.41 to 49.9% of BUSCO genes were found to be absent in clades with 3 or more members (Table 1). Rate of gene absence for clades with 10 or more members were 7.5 to 35.22% (Table 1). The 100 genes having the most pervasive clade-specific gene loss events in the Liliopsida lineage have been depicted in Fig. 4, along with major taxonomic groups where multiple ancestral gene loss events are evident. Gene presence/absence and sequence similarity metrics for all BUSCO genes in the 10 lineages sorted in the order in which they appear along the phylogeny can be obtained in spreadsheet and visual formats from our database website [31].

**Table 1:** Lineage specific ancestral BUSCO gene loss events

Lineage	Resolution level	Number of taxa	BUSCO genes	Genes absent in 3-member clades	Genes absent in 5-member clades	Genes absent in 10-member clades	Genes absent in 20-member clades	Genes absent in 50-member clades
Viridiplantae	Genera	513	425	57 (13.41%)	39 (9.18%)	13 (3.06%)	1 (0.24%)	0 (0%)
Liliopsida	Species	164	3236	549 (16.97%)	279 (8.62%)	143 (4.42%)	0 (0%)	0 (0%)
Eudicots	Species	817	2326	520 (22.36%)	324 (13.93%)	198 (8.51%)	102 (4.39%)	18 (0.77%)
Chlorophyta	Species	83	1519	428 (28.18%)	129 (8.49%)	28 (1.84%)	0 (0%)	0 (0%)
Fungi	Genera	989	758	198 (26.12%)	98 (12.93%)	19 (2.51%)	6 (0.79%)	0 (0%)
Ascomycota	Genera	591	1706	269 (15.77%)	128 (7.5%)	34 (1.99%)	6 (0.35%)	1 (0.06%)
Basidiomycota	Species	782	1764	614 (34.81%)	417 (23.64%)	223 (12.64%)	10 (0.57%)	1 (0.06%)
Metazoa	Genera	2405	954	476 (49.9%)	336 (35.22%)	193 (20.23%)	99 (10.38%)	9 (0.94%)
Arthropoda	Genera	993	1013	220 (21.72%)	133 (13.13%)	58 (5.73%)	24 (2.37%)	0 (0%)
Vertebrata	Genera	1199	3354	941 (28.06%)	466 (13.89%)	220 (6.56%)	89 (2.65%)	16 (0.48%)

Under default BUSCO search conditions, the presence of a moderately divergent homolog would impede the detection of putative gene loss events. For phylogenomic applications, this would create alternate evolutionary histories through processes such as duplications with subsequent gene loss, hybridization or gene flow. Similarly, during BUSCO assessments of new assemblies, undetected gene loss events would inflate estimates of assembly gene content. To assess the prevalence of such complications, we studied the extent of undetected BUSCO gene loss events in our database and their implications for genome quality assessments in the following sections.

### A filtered BUSCO set provides improved assembly assessments

**Fig. 5:**
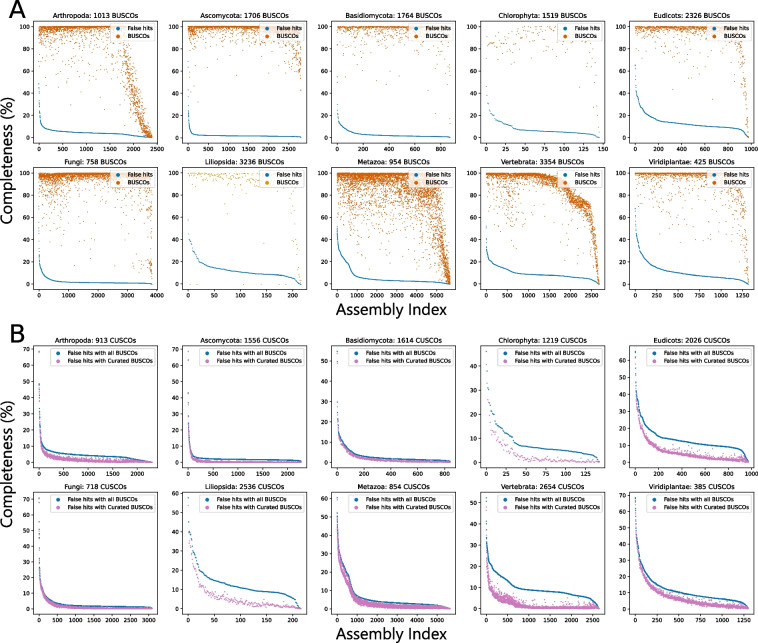
Removal of misannotation-prone BUSCO genes reduces BUSCO misidentification rates.** A** BUSCO genes are misidentified at different rates in different lineages. Values on the horizontal axis are assembly indices after sorting by number of false gene hits. Median fraction of false identification is around 15% for most plants and vertebrates, but noticeably lower in fungi. **B** Only considering our Curated set of BUSCO genes (CUSCOs) reduces false hits in all lineages with marked reductions in Eudicots, Liliopsida, and Vertebrata lineages

Across all 10 lineages, on average 2.25 to 13.33% of BUSCO genes were misidentified in genomes where all BUSCO genes had been removed (Fig. 5A and Table 2). Misidentification implies that a default BUSCO search would not identify divergent copies of these genes and the absence of the identified BUSCO gene in a query assembly would result in the inadvertent identification of the divergent copy. The magnitude of misidentification rates varies by lineage and was observed to be lowest across the fungal assemblies and highest across vertebrate and plant assemblies (Fig. 5A and Table 2). Roughly 10% of BUSCO genes in all 10 lineages were misidentified at a far greater number of assemblies than others (Additional file 1: Figure S10). Assessment of BUSCO completeness with these genes removed resulted in reduced numbers (Table 2) of BUSCO gene misidentifications in all lineages (Fig. 5B). The reduction in false hits was more pronounced in the Vertebrata, Liliopsida, Eudicots, and Chlorophyta lineages (Fig. 5B and Table 2). For clarity, the Curated set of BUSCO genes has been named CUSCOs and the remaining Misannotation-prone BUSCO genes are hereon abbreviated as MUSCOs.

**Table 2:** BUSCO and CUSCO misidentification rates

Lineage	BUSCO completeness (mean)	BUSCO completeness (SD)	CUSCO completeness (mean)	CUSCO completeness (SD)	BUSCO false hits (mean)	BUSCO false hits (SD)	CUSCO false hits (mean)	CUSCO false hits (SD)
Viridiplantae	91.88^a^	15.90	90.67	18.37	11.35	8.69	7.52	8.88
Liliopsida	87.30	24.47	90.06	19.54	12.60	8.01	5.90	7.70
Eudicots	92.37	15.00	91.81	16.52	13.34	7.30	6.35	7.40
Fungi	94.66	12.99	94.93	12.91	2.86	4.01	1.64	4.02
Ascomycota	96.74	6.92	96.84	7.13	2.25	2.97	0.69	3.01
Basidiomycota	95.59	8.08	95.29	9.27	3.00	3.98	2.02	3.86
Metazoa	83.41	23.86	82.32	25.47	6.02	7.44	4.00	6.69
Arthropoda	81.86	29.35	78.86	32.58	4.55	3.92	1.92	3.75
Vertebrata	85.09	19.63	84.72	20.07	9.57	5.12	2.17	3.74
Chlorophyta	86.09	14.29	85.19	16.37	8.12	6.80	3.57	6.09

^a^All numbers are in percentiles

**Fig. 6:**
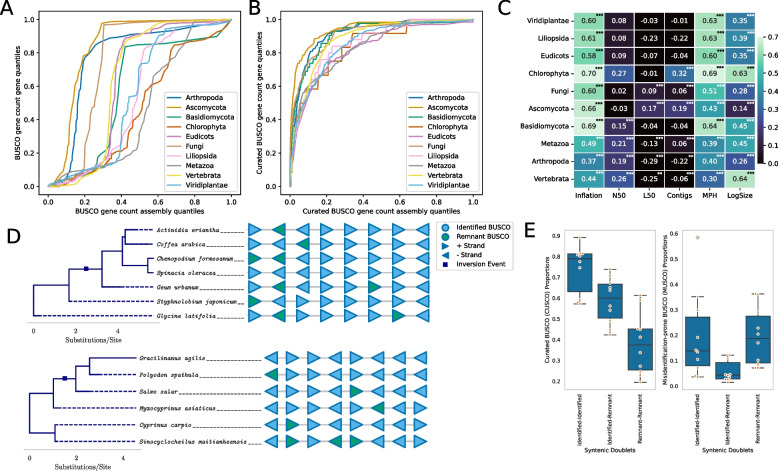
Misidentification events are weighted more towards the identity of the gene rather than assembly and correlate most with assembly complexity and gene content.** A** A graph of gene quantiles against assembly quantiles for false hit counts shows that although the majority of assemblies show some false gene hits, the gene quantiles rise more sharply. **B** Considering only the Curated BUSCO set shifts the assembly quantiles at the lower range towards the genes. Hence, CUSCO genes are misidentified in far fewer assemblies and do not show assembly preferences. **C** False identification rates correlate most with the number of miniProt hits (MPH) and mean BUSCO copy counts (Inflation). Moderate correlation to the log of assembly size is also observed. *: 0.01 < *p*-value < 0.05; **: 0.001 < *p*-value < 0.01; ***: *p*-value < 0.001. **D** Two example blocks of 8 genes conserved beyond the species level for eudicots (top) and vertebrates (bottom) showing misidentified (remnant) BUSCO genes in syntenic order. **E** CUSCO and MUSCO proportions for syntenic doublets with 0, 1, and 2 remnant genes. Remnant proportions gradually recede for CUSCOs, but rise back up in remnant doublets for MUSCOs

We analyzed the incidence of BUSCO misannotations by assembly and gene identity to extrapolate the source of this phenomenon. Gene misannotations were found to be more weighted towards the query gene rather than the query genome assembly (Fig. 6A). Removal of MUSCOs resulted in better assembly assessment metrics and shifted the assembly quantiles of BUSCO misidentification towards the gene quantities (Fig. 6B). Correlation analysis of lineage-wise misannotation rates with assembly metrics revealed that BUSCO gene misidentifications correlated most with the mean number of BUSCO copies in the assembly, a metric we termed inflation (Fig. 6C). Other variables showing the highest correlations were the number of miniProt hits (MPH) and the log of assembly size, being more pronounced in chlorophytes and vertebrates, respectively (Fig. 6C).

Given the observed preponderance of misannotation rates in complex genomes in terms of assembly size, gene hits, and BUSCO inflation (Fig. 6C), we analyzed the syntenic patterns of identified and misidentified BUSCO genes from Compleasm annotations to query potential evolutionary origins. For computational feasibility, all possible permutations of identified and misidentified BUSCO genes in 10 sets of gene blocks harboring up to 10 genes were tested. Gene block analysis revealed that beyond the species level, misidentified BUSCO genes are preserved in syntenic order at the highest rates in the Liliopsida, Viridiplantae, and Eudicots lineages at 4.07%, 3.97%, and 3.78% respectively. The fourth highest rate of syntenic misidentifications was in the Basidiomycota at just 0.88% and the lowest was in Arthropoda at 0.14%. Two such representative gene blocks from the Eudicots and Vertebrata lineages are shown in Fig. 6D top and bottom respectively. This suggests that some misidentified BUSCO genes are remnants of gene duplication events where the syntenic copy became more divergent. Details for all computed gene blocks are available to download from the database website [31]. The syntenic analysis was extended to our complete data set with syntenic gene pairs to determine whether CUSCO and MUSCO genes contained pairs with one and two remnant genes in similar proportions. CUSCO syntenic doublets were progressively found in lower proportions with one and two remnant genes (Fig. 6E). However, MUSCO syntenic doublets appeared in similar proportions with pairs of identified and pairs of remnant genes (Fig. 6E). MUSCO genes are therefore more syntenic in the remnant-remnant configuration compared to CUSCO genes.

### BUSCO collinearity is an indicator of pseudomolecule quality

**Fig. 7:**
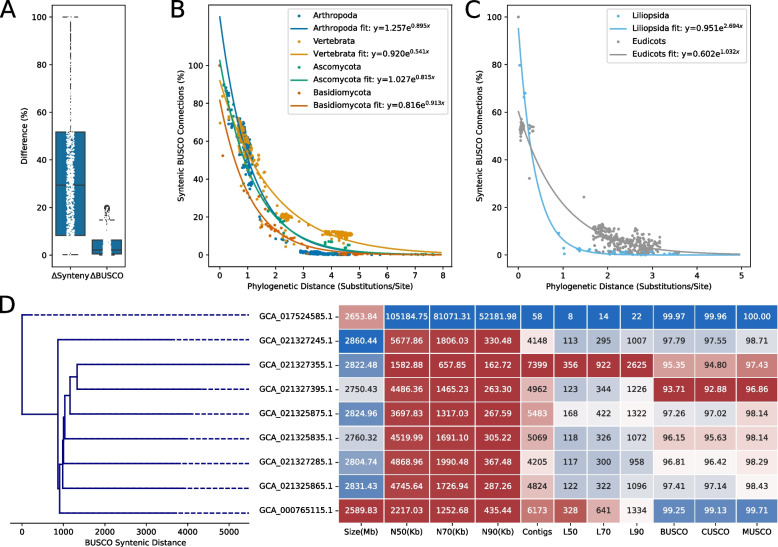
BUSCO syntenic distance offers greater contrast than BUSCO content, decays exponentially with phylogenetic distance and serves as a robust metric to compare closely related assemblies.** A** Boxplot showing differences in BUSCO completeness and BUSCO syntenic distance between 848 pairs of draft versus high-quality assemblies. **B** Exponential decay and curve function of BUSCO syntenic similarity for Arthropoda, Vertebrata, Ascomycota, and Basidiomycota lineages. **C** Exponential decay and curve function for Liliopsida and Eudicots lineages.** D** Eight highly fragmented *Mus musculus* assemblies compared against a highly contiguous assembly (top) through BUSCO syntenic distance and assembly quality metrics

To demonstrate the utility of BUSCO synteny in assembly comparisons, we compiled and compared 848 pairs of genomes of the same species with contrasting quality metrics. The list of compared assembly pairs is provided in Additional file 6: Table S5. We employed an adjusted Intersection Over Union (IoU) metric with BUSCO gene doublets found in the same order and orientation to compare two assemblies. The denominator is adjusted by the difference in the number of contigs such that highly fragmented assemblies with the same gene order and orientation would be syntenically equivalent to highly contiguous assemblies. Hence, the syntenic doublet metric is designed to only capture differences in gene synteny and to not be influenced by varying numbers of contigs in query assemblies (Additional file 1: Figure S11). BUSCO syntenic connections were able to capture far greater contrast in the assembly pairs compared to simply the difference in BUSCO completeness (Fig. 7A). Syntenic BUSCO connections decayed exponentially with phylogenetic distance in our six non-overlapping BUSCO lineages (Fig. 7B and C). We further compiled the 40 least contiguous NCBI assemblies of *Oryza sativa*, *Mus musculus*, *Drosophila melanogaster*, *Ovis aries*, and *Arabidopsis thaliana* to represent the BUSCO syntenic distance between the assemblies as a dendrogram. Metrics for the full set of assemblies are provided in Additional file 1: Figures S12, S13, S14, S15, and S16 respectively. An example of a dendogram with 8 fragmented *Mus musculus* assemblies and a highly contiguous reference assembly is shown in Fig. 7D. Less contiguous assemblies were found to be at greater syntenic distances to the higher-quality assembly, implying greater numbers of BUSCO misidentification events or more extensive misassemblies.

**Fig. 8:**
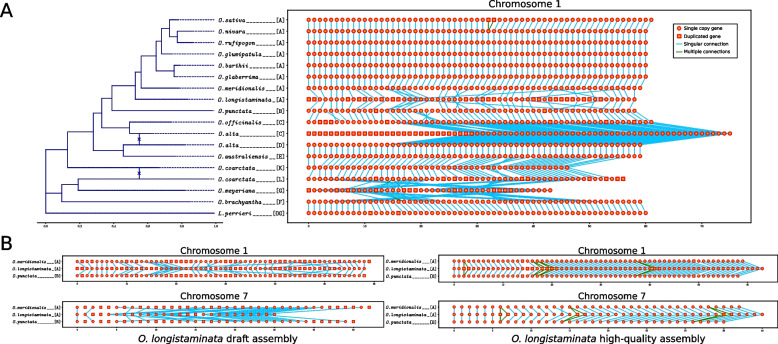
Phylogenetic and syntenic information improve assembly assessments.** A** BUSCOs in chromosome 1 of *Oryza longistaminata* and *O. meyeriana* assemblies are less syntenic to sister taxa. A chromosomal translocation event from chromosome 3 to 1 in *O. alta* subgenome C is also visualized. **B** Assessment of an improved *O. longistaminata* assembly reveals that BUSCO genes were either misidentified or contigs were scaffolded poorly in the inferior assembly

To further assess how BUSCO synteny can indicate assembly quality, we visualized chromosome-wise BUSCO collinearity in a set of *Oryza* assemblies as a case study. The *Oryza* genus is genomically well characterized with several state-of the-art chromosome level assemblies [12]. We demonstrate with a draft assembly (GenBank ID: GCA_009805545.1) and a high-quality assembly of *Oryza longistaminata* [38] that BUSCO synteny can provide greater contrast between assemblies of varying quality compared to BUSCO metrics alone (Fig. 8). Between the two *O. longistaminata* assemblies, although the number of curated BUSCO genes identified was comparable (98.82% and 93.17%), BUSCO collinearity was not preserved across the closely related sister taxa within the genus (Fig. 8). These observed syntenic deviations are quantified by our adjusted IoU metric based on BUSCO gene connections (Additional file 1: Figure S11) and the syntenic distance between the two *O. longistaminata* assemblies was 82.25%. The full set of chromosomes for this test case is available on the database website [31]. The phyca software package [32] allows users to similarly compare and visualize syntenic distances between assemblies and query genomes.

## Discussion

Here, we presented our studies across three facets. First, we determined the prevalence of BUSCO gene variations by taxonomy through the compilation of available plant, fungi and animal genomes in the public domain. Second, we optimized site conditions for consistent phylogenomic reconstructions by maximizing taxonomic congruity and minimizing tree set variability. We then created large genome-scale phylogenies under the best determined conditions for 10 major BUSCO lineages. Third, we provided evidence for BUSCO misannotations with the current software defaults and filtered a curated set of BUSCO genes for better genome quality assessments. To mitigate the effects of BUSCO misannotations during assembly evaluations, we described a novel method of comparing assemblies with BUSCO synteny that provides much better contrast for closely related assemblies of varying quality.

### BUSCO completeness and copy number variations

Universal genes have been instrumental for querying gene space completeness and assembly quality [7]. Our results show that the evolutionary history of a genome influences its BUSCO score and that this influence is prevalent in many taxonomic groups rather than just a few [10]. It was also observed that some groups vary more dramatically than others in BUSCO metrics (Additional file 2: Table S1). Therefore, for assemblies from early diverging groups with few extant taxa or available genomes, BUSCO genes may provide an inadequate representation of gene space completeness. Given these observations, we propose that it is necessary to consider the evolutionary history of related taxa when evaluating the gene content of new genome assemblies.

Assembly gene content is influenced drastically by evolutionary history. Polyploid organisms are known for being able to maintain multiple sets of single-copy orthologs [12] and genomes fractionate at varying rates post-duplication [39]. It is likely that groups that were found to harbor large sets of duplicated BUSCO genes in haploid assemblies have either experienced recent whole-genome duplication events or have adjusted their gene regulation to accommodate an inflated complement of some single-copy orthologs. The set of genes that are more likely to be misidentified (Additional file 1: Figure S10) are likely tolerated more in genomes at greater copy numbers. This is supported by the high correlation of gene misannotations to the BUSCO inflation metric shown in Fig. 6C and the preservation of some syntenic remnant genes across large phylogenetic distances (Figs. 6D and E). It is probable that misannotation-prone genes duplicated and subsequently functionalized in ancient ancestral genomes multiple times [40]. Some of the duplicated copies may have taken up important functions that prevented the sequences from diverging drastically and the shared homology is now responsible for the observed false hits. The availability of a consolidated database of BUSCO results from public genomes allows researchers to derive meaningful copy number expectations for BUSCO genes in new assemblies based on evolutionary history.

### Decoupling aligned sites from gene features and a case for fast evolving columns

Likelihood estimation in phylogenetics assumes that all sites evolve independently [41]. Phylogenetic methods often specify invariant sites [42], discrete rate categories [43], and sequence partitions [44] to address among-site heterogeneity [41]. However, site interdependence [45] is generally not accounted for by available methods [46]. We assumed that unique amino acid counts in aligned columns could serve as a proxy for evolutionary rate at that site and filtering sites by evolutionary rate would decouple sites from intragenic evolutionary influences. In practice, researchers often select fast evolving sites for dense phylogenies [47] and conversely, for deep phylogenies, they tend to use slowly evolving sites to optimize information content in the alignment [19]. Our study broadly highlights the practical effects of rate variation and alignment information content on tree reconstruction. Tan et al. [48] demonstrated that alignment filtering negatively impacts tree accuracy. Our results show that it may be possible to overcome such effects by using multilocus site patterns with more unique characters, as it can mimic the effects of using longer alignments (Fig. 2). Rosenberg and Kumar [49] showed that the number of sites have greater effect on tree accuracy compared to substitution rates. On the contrary, our results show that when an adequate number of sites are sampled (Fig. 2C), site evolutionary rate has a greater effect on tree accuracy in terms of taxonomic congruity. In our studies, higher rate sites were generally found to produce better trees and there was minimal hindrance caused by long-branch attraction biases (Fig. 2B and C).

Slow evolving sites have been favored throughout the history of molecular phylogenetics [50]. Slow-fast analysis was popularized for phylogenetic reconstructions in the context of substitution saturation and long-branch biases [51, 52]. Similarly, chi-squared tests are employed to detect compositional heterogeneity in alignments [53, 54]. The primary goal of these analyses has been to identify and prune fast evolving sites to improve phylogenies [50]. Such practices have recently been perceived with scrutiny [55, 56] has shown that fast evolving alignment sites can be highly informative. We show in Fig. 2 (and Additional file 5: Table S4) that higher rate sites improve taxonomic concordance across almost all 543 families tested, and always increase tree set consistencies (Additional file 1: Figure S8) compared to lower rate sites. Therefore, contrary to common practices, our results suggest that with adequate taxon sampling, faster rates for protein characters may produce more accurate phylogenies regardless of node depth.

### Phylogenies within the kingdom Fungi and recalcitrant evolutionary histories

Some taxonomic classifications in the fungal domain are based on molecular ITS (Internal Transcribed Spacer) data [57]. Although ITS-based primers are commonly used for phylogenetic placement, the drawbacks of ITS sequences are apparent. RNA code has fewer letters than protein code and the ITS sequences are much shorter than most protein coding genes. Further, rRNA genes appear in large copy numbers [58, 59] making them amenable to multiple evolutionary histories at greater divergence times. In contrast, single-copy orthologs exist under dosage restraints and this generally prevents copy number variations from persisting throughout evolutionary timescales [39]. Additionally, sampling greater numbers of taxa generally has a strong positive effect on phylogenetic accuracy [60] and BUSCO genes offer the means to include highly divergent clades. For these reasons, it is reasonable that BUSCO genes would be able to resolve deeper phylogenies with greater precision than ITS sequences.

We found taxonomic classifications to be more obscure for the kingdom fungi. Although tree entropy at the termini reduced by about 50% (Additional file 1: Figure S8), we did not observe the same level of gradual reductions in the variance of monophyletic counts as seen from plants and higher animals (Additional file 1: Figure S7). One likely explanation for these complications is their significantly higher rate of evolution and shorter generation times compared to other clades [61, 62]. This can be seen in the greater fraction of high-rate sites shown in the state frequency spectra in Additional file 1: Figure S3. This effect in conjunction with their compact genome sizes, relatively higher rates of gene flow [63] and very short generation times compared to higher eukaryotes makes the accurate reconstruction of fungal evolutionary histories challenging. Despite these challenges, the fungal families did follow the same trend as the higher eukaryotes in response to increasing evolutionary rates in Figs. 2B and C, albeit a greater fraction of families seemed to have members descended from more than one most recent common ancestor. The greater fraction of non-monophyletic groups could be an artifact of the limitations of the standard ITS-based classification scheme. These views are supported by a 9.72% observed higher fraction of monophyly in the higher fungi, basidiomycetes compared to the lower fungal phyla, ascomycetes (Additional file 5: Table S4). Furthermore, compared to the other three lineages tested, ascomycetes and basidiomycetes show noticeably greater numbers of monophyletic groups with alignments of slowly evolving sites (Additional file 1: Figure S4). One cause behind this could be higher rates of alignment errors in more distantly related taxa. In this regard, we did not consider the consistently reproduced alignments in Additional file 1: Figure S2 to be infallible since they are biased by the heuristics of multiple-sequence alignment algorithms [25]. Additionally, the higher range of monophyly in shorter alignments (Additional file 1: Figure S4) could be explained by ITS-derived taxonomic classifications since those alignments resemble ITS alignments better in terms of length, slower rates of evolution, and overall information content. Because of these ambiguities, assessments of phylogenetic accuracy for fungal lineages remain a formidable challenge.

### BUSCO provides a standard for genome-scale phylogenies

At present, both concatenated and coalescent phylogenies are used in practice [18, 29]. The multispecies coalescent model accounts for incomplete lineage sorting to resolve ancestral relationships in higher taxa speciating from large populations. Jian et al. [64] showed that the multispecies coalescent outperforms concatenation across a range of metazoan groups. Our results suggest that such differences may vary significantly by lineage (Fig. 3) and are marginal in terms of taxonomic congruity (Additional file 1: Figure S9). In terms of gene concordance factors, our concatenation trees had lower variances in all cases and higher means in 3 out of 5 tested lineages. It is also important to note that the total number of sites in the coalescent trees were far greater than the concatenated trees since up to 75 whole genes were combined. For comparison, the vertebrate tree likelihoods were still improving at the 10,000 site count mark (Additional file 1: Figure S6). We propose that when there is adequate information content in the alignments, the high dimensional likelihood surface flattens out harboring several vicinal and localized peaks and valleys. This results in the distribution of alternate topologies with varying model likelihoods spread out within a range of monophyly counts in the correlation plots shown in Additional file 1: Figure S6. We thus conclude that the multispecies coalescent offers a powerful framework, but results should still be interpreted with caution, and our BUSCO concatenation method offers a robust alternative when suitable. Site-filtered BUSCO concatenation trees may be preferred when studying the ancestral history of divergent or monotypic taxa using whole genome data.

The search space for phylogenetic trees grows faster than exponentials with increasing numbers of terminal nodes [41]. Our smallest tested tree had 592 terminal nodes which equates to a search space of $\frac{((2\times 592)-5)! }{{2}^{(592-3)}\times (592-3)!}=2.12\times {10}^{1556}$. This high number of taxa makes the tree space numerically intractable even with the best available heuristics. The exact same tree topology was never reproduced in our results under any condition. From our evaluations of the tree distributions, we suggest that (1) consistent reconstruction of a greater number of groups as monophyletic offers support for internal nodes and (2) reduced terminal variability in tree distributions provides confidence for accuracy of overall tree topology. Combined, ancestral histories reconstructed from our method of sampling high-rate sites from whole-genome BUSCO data should be deemed more reliable than ITS or gene trees, and on par with coalescent-based trees. Out of the tested families, 39 clades had undetermined monophyly status (Additional file 5: Table S4) and users of phyca must be cautious about directly interpreting their evolutionary histories from our tree sets. It is important to be aware that with large datasets, model inadequacies [65] could result in erroneous topologies having high support values. It is therefore possible that for any individual taxa or clade, the reduced terminal variability in our tree sets may have reinforced erroneous placements. We recommend that researchers with more nuanced evolutionary questions should consider rebuilding subtrees within their clade of interest. For this purpose, phyca provides a user-friendly implementation of our proposed methods to construct phylogenies from user defined sets of query taxa.

### Shortcomings of homology-based and probabilistic gene predictions

BUSCO has been the unrivaled standard for gene space completeness tests since 2019 [66]. BUSCO relies on sequence homology searches through sequence alignments and subsequent refinement of search results by trained hidden Markov models [7]. In general, alignment-based methods for gene identification are employed using arbitrary cutoffs [67] and probabilistic models are used with empirically trained probabilities [25, 68]. BUSCO gene prediction by Compleasm [69], a better implementation of BUSCO, starts with a miniProt [70] search that is restricted to report duplicate genes only if the alignment score is at least 95% of the best alignment. Compleasm has four additional threshold parameters for secondary hits, gene identity, fraction, and completeness respectively. These thresholds have been empirically optimized by the developers to maximize precision and recall [69]. Almost all user-reported BUSCO results are reported based on default parameters [12, 14, 15, 33, 34]. Readjustment of these parameters would adversely alter the preoptimized tunings, and for experimental explorations, there would be an inordinate number of permutations to consider. Our method of removing genes and rerunning under default settings mimics the effect of putative gene loss events. Our analysis of false positive hits revealed a set of less reliable BUSCO genes with a significantly higher propensity of being misannotated (Additional file 1: Figure S10). We surmise that for gene predictions there may be no “one glove fits all” method that will work for all genes across all possible lineages. With this view in mind, integrative approaches have been suggested in the past to improve gene prediction accuracies [71]. We conclude that putative gene prediction is a tricky endeavor and demonstrate in Fig. 5B and Table 2 that omitting the less reliable genes from the BUSCO gene set improves precision without compromising recall.

## Conclusions

Universal orthologs are critical inferential tools for evolutionary genomic research. To improve the utilization of BUSCO genes in this field, we first compiled and comprehensively analyzed their presence and copy number variations within the expansive higher eukaryotic domain. Based on our findings, we suggest that evolutionary histories must be considered for proper interpretation of BUSCO completeness metrics. Second, we determined the extent to which the ancestral histories of major eukaryotic lineages could be resolved through universal single-copy orthologs. Our results imply that columns evolving at higher rates in alignments of protein characters are more robust for deep phylogenomic reconstructions. We described a novel way to consider phylogenetic accuracy using taxonomy and a simplified way to express tree set variability by enumerating terminal leaf bifurcations. In light of our findings, we produced the largest unified nuclear genome-based phylogenies for 10 major taxonomic groups in the plant, fungi and animal kingdoms to date. Within these phylogenies, we highlighted familial clades that were consistently reconstructed as monophyletic with respect to their taxonomic labels and distinguished clades that demonstrated more recalcitrant ancestral histories. Finally, our database yielded a filtered set of BUSCO orthologs that provide a better representation of assembly gene content compared to the standard BUSCO search. We showed that more robust evaluation of genome quality can be attained through the incorporation of BUSCO syntenic information from related assemblies. Our processed data and tools have been made easily accessible for robust phylogenomic reconstructions, rapid placement of query assemblies by appending BUSCOs to large, precomputed alignments and for deriving phylogenetically informed assembly quality evaluations.

## Methods

### Database compilation and classification

Metadata for plant, fungi, and animal genome assemblies were sourced from the NCBI genome database [72] accessed on January 14, 2024. Assemblies flagged by NCBI as partial and contaminated were not used. Special characters (\'()-/#: = + []) were removed from organism names to avoid software errors during automation. The assembly metadata were sorted by level of assembly set by NCBI (complete, chromosome, scaffold, contig), date of release (newest to oldest), and assembly size (largest to smallest) respectively. Only the top entry for identical organism names was kept. Batch downloads were executed using the cURL application (www.curl.se). The NCBItax2lin software (https://github.com/zyxue/ncbitax2lin) was used to assign taxonomic classifications at the phylum, class, order, family, and genus levels to the assemblies. The Mann–Whitney test was used to test the hypotheses of whether assemblies within a taxonomic group had a significantly different mean for a metric compared to all assemblies in the BUSCO lineage. A Bonferroni correction of $\frac{0.05}{2\times(total\;assembly\;count)}$ was carried out to determine the *p*-value cutoff thresholds.

### Finding and aligning universal orthologs

Searches for universal orthologs was executed using Compleasm version 0.2.5 [69] with OrthoDB version 10 reference sequences [8] for the Viridiplantae, Chlorophyta, Liliopsida, Eudicots, Fungi, Ascomycota, Basidiomycota, Metazoa, Arthropoda, and Vertebrata lineages using the default settings. For duplicated universal single-copy orthologs, the ortholog that was more syntenic with the database was selected. Gene copies sharing adjacent BUSCO orthologs at greater frequency within the database were defined as more syntenic. For equally syntenic duplicates, the gene with greater sequence identity was retained. Assemblies that did not contain 90% of the BUSCO orthologs in each lineage were included in the database but dropped from the subsequent phylogenetic analysis for suboptimal quality. Protein characters from all identified orthologs for each gene in each lineage were aligned using MUSCLE version 5.1 [25]. Depending on lineage, approximately 200,000 (Viridiplantae) to 2,000,000 (Vertebrata) total sites were aligned. Alignments for the Viridiplantae, Fungi, Metazoa, and Arthropoda lineages were done with 16 total combinations of four parameter perturbations and four guide tree permutations to create a stratified ensemble of multiple sequence alignments described in Edgar [25]. Confidence for each column in the alignment was computed using the addconfseq flag in MUSCLE v5.

### Phylogenetic assessment

For Eudicots, Ascomycota, Basidiomycota, Arthropoda, and Vertebrata lineages, aligned sites were filtered by the number of unique amino acids in the column as a proxy for rate of evolution at that site. For 2 to 15 unique amino acids, we selected between 1000 and 20,000 sites at 1000 site increments. Only the exact number of unique amino acids was included in each rate category. This resulted in a total of 14 × 20 = 280 alignments per lineage. For the Arthropoda lineage, we could only select up to 14,000 sites per category because of the relatively lower number of aligned sites. We had 14 × 14 = 196 total alignments for arthropods. Assemblies that had fewer than 90% BUSCO genes and aligned sites that comprised more than 10% gaps were removed. IQ-TREE version 2.1.2 [46] was used with default settings including built-in ModelFinder2 [73] to create maximum likelihood trees for every alignment. There were 198 total nuclear substitution models to test including permutations of alternate character frequencies and rate heterogeneity. A total of 280 × 4 + 196 = 1316 individual trees were created and tested in this step (196 for arthropods and 280 for the 4 remaining lineages). Trees were assessed for taxonomic congruity by counting the number of families that descended monophyletically from a common ancestor. For the terminal and central rates 2, 8, and 14, five sets of alignments were sampled for site counts 1000, 5000, and 10,000. We carried out 10 independent searches on the tree space for each alignment with a different random seed, resulting in a total of 10 × 5 = 50 trees for 3 × 3 = 9 conditions in 5 lineages. The total number of trees at this stage was 50 × 9 × 5 = 2250. Each individual tree was assessed for congruity by counting the number of monophyletic families. Taxonomic trees were created from NCBI taxonomic data using BioNick version 0.0.8. Robinson-Foulds distances were computed using the TreeCmp software (https://github.com/TreeCmp/TreeCmp). The set of trees in each condition was assessed for entropy or degree of variation at the terminal leaves by counting the total number of unique terminal bifurcations in the set. The 5 alignment sets at site rate 8 and site count 5000 were used to compute likelihoods for all 2250 trees using IQ-TREE. The mean likelihood score of 5 alignments was used as the likelihood for each individual tree. Gene trees were created for 100 BUSCO genes with the lowest number of missing taxa and highest gene length in the Eudicots, Ascomycota, Basidiomycota, Arthropoda, and Vertebrata lineages. From 5 to 75 genes were selected with increments of 5 genes at random to create 15 coalescent trees under the multi-species coalescent model in Astral-pro3 version 1.19.3.5 [74]. Gene concordance factors were calculated from 100 BUSCO gene trees using IQ-TREE version 2.1.2 [46].

### Identifying BUSCO gene loss events

Phylogenies were created for all 10 lineages at either specific or generic levels depending on the number of available taxa and required computational load using 10,000 sites having exactly 14 amino acid characters. For every internal node in each phylogeny, BioNick version 0.0.8 was used to test whether all descendants were missing a particular BUSCO gene. Internal nodes with three or more descendants supporting an ancestral gene loss event were considered. Genes were sorted by the total number descendant nodes with supported loss events, exported and visualized using BioNick version 0.0.8 and the Python Matplotlib library [75]. Values for gene identity and fraction were obtained from Compleasm annotations.

### Assessing misidentified BUSCOs

For BUSCO misidentification studies, all single and duplicate BUSCO genes identified by Compleasm were first removed using scripts available on the phyca GitHub page and Compleasm was rerun on the genome set. Genes found in fragments were not considered. The curated BUSCO gene set was selected manually by looking at the frequency at which each BUSCO gene was misidentified. For each assembly, genome inflation was defined as the average number of times the BUSCO gene set was found in the assembly. Polyploid genomes shown in Additional file 1: Figure S1 were labeled manually according to literature through searches done by the species names. Assembly level for chromosome scale assemblies was determined by the labels assigned to the pseudomolecules. Pearson’s correlation coefficient was used for correlation analysis of misannotated BUSCOs to genome properties. The number of miniProt hits (MPH) for correlation analysis was obtained directly from Compleasm. *P*-values for correlation tests were computed using the SciPy package [76].

All gene syntenic analyses were based on default Compleasm genome annotations. Gene blocks were traced with all possible permutations of identified and remnant BUSCO genes up to 11 genes in length using phyca scripts. An identified BUSCO gene was defined as a gene that was properly annotated by Compleasm and a remnant BUSCO gene was defined as a gene that would supplant an identified BUSCO gene in a Compleasm run following the deletion of all identified BUSCO genes in the genome. To compute CUSCO and MUSCO proportions, Remnant-Identified gene doublets were considered syntenic when they were matched in gene identity and orientation by a Identified-Identified doublet within the same lineage. Remnant-Remnant gene doublets were considered syntenic when they were matched by either a Remnant-Identified doublet or an Identified-Identified doublet. For each set of BUSCO doublets, fraction of doublets where both genes were CUSCO genes was defined as the CUSCO proportion and the fraction of doublets where both genes were MUSCO genes were defined as MUSCO proportions.

For comparisons of BUSCO gene content and syntenic distance, two assemblies of the highest and lowest N50 were selected for organisms with more than one available genome assembly from NCBI Genome. Only pairs where the difference in N50 was greater than 200 Kb were considered. Assemblies with an N50 of less than 1 Mb or less than 80% BUSCO content were filtered out. Syntenic distance and distance matrices were computed by phyca. The adjusted Intersection over Union (IoU) metric was computed as follows:

$$distance=\frac{Shared\;BUSCO\;gene\;pairs\;by\;gene\;identity\;and\;orientation\;(I)}{Total\;BUSCO\;gene\;pairs\;by\;identity\;and\;orientation\;\left(U\right)-difference\;in\;the\;number\;of\;contigs\;(\Delta contig)}$$

where subtraction of the difference in contigs numbers is our proposed adjustment to a standard IoU metric. Exponential curves were fit using the curve_fit function from SciPy [76] version 1.14.1. Distance matrices were converted to newick trees using scikit-bio version 0.6.2 (https://scikit.bio).

The *Oryza alta* assembly was from Yu et al. [77], and *Oryza coarctata* was from Fornasiero et al. [12]. Pseudomolecules of two subgenomes of the polyploid *Oryza* species were separated through their sequence headers. All dendograms and cladograms were created using BioNick version 0.0.3 (https://pypi.org/project/BioNick/). The phyca website uses phylotree.js (https://phylotree.hyphy.org/) for dynamic tree visualizations.

### Database and software limitations and maintenance

Our database and analyses comprise only the 10 most genomically well-represented eukaryotic BUSCO lineages. Similarly, the phyca toolset only supports these 10 major BUSCO lineages. The genome database is intended for eukaryotic genomes and prokaryotes will not be included. The phyca website and database will be updated biennially over the lifetime of the EAGI host server located at the University of Arizona. A 2025 database update is currently underway. The software phyca and BioNick will be maintained on GitHub to support subsequent versions of Compleasm [69] and the OrthoDB [8] database.

## Supplementary Information

Additional file 1: Supplementary Text. Fig. S1. Mean BUSCO copy numbers correlate with heterozygous and polyploid genomes assembled at higher levels. Fig. S2. Multiple sequence alignments vary based on alignment algorithm parameters. Fig. S3. Amino acid state frequency spectra for 10 lineages. Fig. S4. Taxonomic concordance of trees as a function of site evolution rates and alignment lengths. Fig. S5. Robinson-Foulds distances to taxonomic trees as a measure of taxonomic concordance. Fig. S6. Correlations between tree likelihood and taxonomic concordance under different rate and site configurations. Fig. S7. Variations in taxonomic concordance within trees created under the same rate and site conditions. Fig. S8. Counts of unique terminal leaf bifurcations in 9 conditions for 5 tested lineages. Fig. S9. BUSCO concatenation and coalescent trees have similar accuracy in terms of family monophyly counts. Fig. S10. Exclusion of BUSCO genes that are misidentified at far greater propensity than others. Fig. S11. Demonstration of using the proposed BUSCO syntenic identity metric and how it is affected by variations in assembly contiguity, insertions, deletions, inversion and fragmentation in both assemblies. Fig. S12. BUSCO synteny used as a distance metric to represent the magnitude of differences between 41 *Oryza sativa* assemblies. Fig. S13. BUSCO synteny used as a distance metric to represent the magnitude of differences between 41 *Mus musculus* assemblies. Fig. S14. BUSCO synteny used as a distance metric to represent the magnitude of differences between 41 *Drosophila melanogaster* assemblies. Fig. S15. BUSCO synteny used as a distance metric to represent the magnitude of differences between 41 *Ovis aries* assemblies. Fig. S16. BUSCO synteny used as a distance metric to represent the magnitude of differences between 41 *Arabidopsis thaliana* assemblies.

Additional file 2: Table S1. BUSCO metrics for 2,606 taxonomic groups across 10 BUSCO lineages.

Additional file 3: Table S2. Substitution models with the highest likelihood under all tested rate and site profiles.

Additional file 4: Table S3. Modelfinder2 results for substitution models for tested rate and site profiles.

Additional file 5: Table S4. Familywise incidence of monophyly under different rate and site conditions.

Additional file 6: Table S5. Assembly metadata from pairwise assembly comparisons.

## Data Availability

This study uses compiled genomic data from the public NCBI genome database. No new sequence data were generated by the authors. All bulk and processed research data have been made available at https://www.phyca.org/data.html. The scripts and code used to analyze the bulk data and prepare manuscript figures are available at https://github.com/DeadlineWasYesterday/UniPhy. The phyca and BioNick software tools developed in this study are open-source and available at https://github.com/DeadlineWasYesterday/phyca and https://github.com/DeadlineWasYesterday/BioNick.

## Abbreviations

BUSCO: Benchmarking Universal Single Copy Orthologs
CUSCO: Curated Universal Single Copy Orthologs
MUSCO: Misannotation-prone Universal Single Copy Orthologs
NCBI: National Center for Biotechnology Information
LG: Le-Gascuel (substitution model)
JTT: Jones-Taylor-Thornton (substitution model)
BIC: Bayesian Information Criterion
RF: Robinson-Foulds (distance)
MPH: MiniProt Hits
ITS: Internal Transcribed Spacer
